# Book Notices

**Published:** 1878-06-20

**Authors:** 


### BOOK NOTICES.

THE AMERICAN ANTIQUARIAN—A journal of Anthropology, devoted especially to History, Ethnology and Archœology — Illustrated. Published quarterly, by Brooks & Schinkle, Cleveland, Ohio.

This journal is ably edited, and pertains to a very attractive and interesting field of scienttfic study.

LAPERO-ELYTROTOMY, a substitute for the Cæsarean Section, by S. Gaillard Thomas, M.D., New York.

In this operation the incision is made above Poupart's ligament, extending from the spine of the pubis to the anterior superior spinous process of the illium. The peritoneum is turned aside, and an incision made through the vaginal wall near its junction with the cervix uteri, through which it is practicable to deliver the child. Five cases are reported in the above paper, in which the number of mothers surviving are three, and number of children four.

SOUTHERN HOMES illustrated, being a collection of buildings recently erected by A. C. Bruce, Architect and Superintendent, Knoxville, Tenn. A neat little work of 60 pages, to be used, as the Author remarks, as "suggestive studies in practical and original designs by which those who comtemplate building may profit, and enable themselves to build beautiful and comfortable homes."

The author is a practical architect, of much experience, and those designing to build and who desire plans, specification, etc., would do well to communicate with him at Knoxville, Tenn.

FLUID EXTRACTS by repercolation, by Edward R. Squibb, M.D., Brooklyn, New York.

A pamphlet of forty-three pages, containing important suggestions in pharmacy and showing an improved method of repercolation.

A new treatment of Spinal diseases, by Meigs Case, M.D., Oneonta, New York. A pamphlet of interest, containing valuable suggestions.

NEW MEDICINES and their Special Therapeutics, by I. J. M. Goss, A. M. M.D., of Marietta, Ga., author of Materia Medica and Therapeutics, etc. Chas. E. Ware & Co., St. Louis, printers.

This work, the author states, contains a concise notice of most of the new remedies, and the more direct action of some of the old ones. Anything relating to the properties of new medical agents, and especially our indigenous remedies, will attract attention and intererst. The author gives notice of a forthcoming larger work on the same subject; also a work on practice, by which we infer that he is a vigorous writer and an energetic man. We have not found time to examine the work critically.

Observations on Practice, Surgery, Gyneocology, and especially Obstetrics, by Geo. B. Walker, M.D., Professor of Obstetrics in the Medical College of Evansville.

A paper containing many useful and practical suggestions.

---

S. H. KENNEDY's Concentrated Extract of Pinus Canadensis.

See his advertisement.

See advertisement headed Special Notice, T. Crosby, chemist, New York, relative to Vitalized Phosphates as Brain and Nerve Food.

UNIVERSITY OF THE CITY OF NEW YORK—Medical Department. Examine the advertisement.

COLLEGE OF PHYSICIANS AND SURGEONS, NEW YORK. Advertisement in present number of RECORD.

SAMUELS, PEMBERTON & REYNOLDS, DRUGGISTS, ATLANTA, GA.— This enterprising firm make an addendum to their advertisement in our present issue, giving notice of their wholesale agency for the house of Merrell, Thorp & Lloyd, of Cincinnati.

BATTLE & Co., chemists, of St. Louis, advertise in this issue BROMIDEA, which they style the "hypnotic par excellence." The combination seems admirable, and the profession should give it a trial.

MERRELL, THORP & LLOYD.—We invite attention to the card of Messrs. Merrell, Thorp & Lloyd, Cincinnati, in this issue of our journal, addressed "To Physicians and Druggists." We return thanks to these gentlemen for beautiful samples of fluid extracts, which have just come to hand, to wit—Gelseminum, Veburnum Prunifolium, Hyosceamus, Belladonna, Aconite, etc. We have already tested a number of their preparations, and feel safe in saying that they are pure, strong, and reliable. As will be seen from their card above referred to, they have established a Southern depot at the drug establishment of Pemberton, Samuels and Reynolds, Atlanta, Ga., where their medicines can be had at wholesale rates.

TO PHYSICIANS AND DRUGGISTS.

